# Chéloïde géante retro-auriculaire droite post-traumatique

**DOI:** 10.11604/pamj.2019.33.62.16912

**Published:** 2019-05-28

**Authors:** Sokona Touré, Mahamadou Mallé

**Affiliations:** 1 Faculté de Médecine et d’Odontostomatologie de Bamako, Bamako, Mali; 2 Service de Radiologie, Hôpital Régional de Gao, Gao, Mali

**Keywords:** Chéloïde, traumatique, retro-auriculaire, Keloid, traumatic, retroauricular

## Abstract

Keloid is a benign fibroblastic tumor which is most often secondary to a scar, but it can occur spontaneously in a subject with black skin. We report the case of a 19-year old patient, electrician, presenting with giant right retroauricular mass evolving over two years. For some time, the patient has experienced a feeling of heaviness on the pavilion of the right ear. The lesion had manifested as a hard, pruritic, non-painful patch gradually increasing in size. The patient had received several topical and antiseptics treatments without any effect. He had a history of traumatic injury in the right retroauricular sulcus. A month after, pruritic hypertrophic scar occurred. Physical examination showed voluminous hard, fixed, retroauricular tumor (10 × 6 cm) with normal skin color, non-painful on movement (A). Its base was sessile. The remainder of the physical examination was normal. Ultrasound showed little vascularized skin tumor of the right retroauricular sulcus with more or less sharp margins, measuring 9 x 5 cm. Two diagnoses were suspected: post-traumatic keloid (most likely) and Darier Ferrand dermatofibrosarcoma. Biopsy was performed and histological examination showed keloid. The patient underwent intramarginal excision (B). The surgical specimens weighed 75 grams (C). The postoperative course was simple with disappearance of the feeling of heaviness.

## Image en médecine

Une chéloïde c'est une tumeur fibroblastique bénigne le plus souvent secondaire à une cicatrice, mais peut être spontanée chez un sujet à peau noire. Nous rapportons le cas d'un patient âgé de 19 ans, électricien qui a consulté pour une masse géante retro-auriculaire droite, évoluant depuis deux ans. Depuis quelques temps, le patient ressent une sensation de pesanteur sur le pavillon de l'oreille droite. La lésion avait débuté par une plaque dure, prurigineuse, non douloureuse qui a progressivement augmenté de taille. Le patient avait reçu à plusieurs reprises des traitements topiques et antiseptiques sans effet. Il y'avait une notion de blessure traumatique dans le sillon retro-auriculaire droit. Un mois après, apparait une cicatrice hypertrophique prurigineuse. L'examen physique trouvait une tumeur retro-auriculaire de grande taille (10 × 6 cm), couleur peau normale, dure, fixe, non douloureuse à la mobilisation. La base était sessile. Le reste de l'examen était normal. L'échographie à montre une tumeur cutanée peu vascularisée du sillon retro-auriculaire droit de contours plus ou moyens nets, mesurant (9 x 5 cm). Nous avions évoqué deux hypothèses diagnostiques: une chéloïde post-traumatique le plus probable et le dermato-fibrosarcome de Darier Ferrand. Une biopsie a été réalisée et l'histologie a conclu à une chéloïde. La conduite a été l'exérèse chirurgicale intra-marginale. Les pièces opératoires pesaient 75 grammes. Les suites opératoires étaient simples avec disparition de la pesanteur.

**Figure 1:**
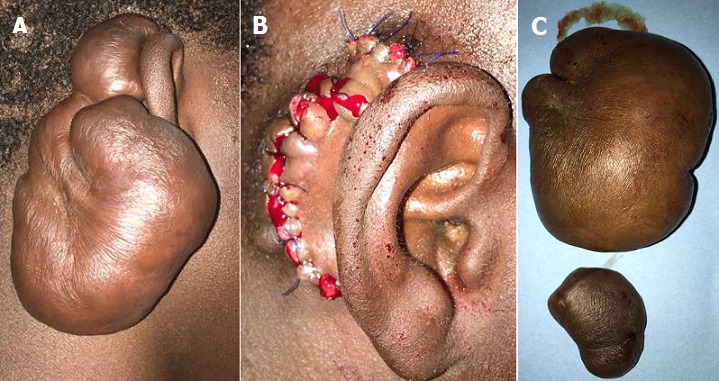
A) chéloïde géante retro-auriculaire droite chez un jeune de 19ans; B) plaie post opératoire; C) pièces opératoires

